# Translating research findings to clinical nursing practice

**DOI:** 10.1111/jocn.13586

**Published:** 2016-11-22

**Authors:** Kate Curtis, Margaret Fry, Ramon Z Shaban, Julie Considine

**Affiliations:** ^1^ Sydney Nursing SchoolUniversity of SydneyCamperdownNSWAustralia; ^2^ Trauma ServiceSt George HospitalKogarahNSWAustralia; ^3^ St George and Sutherland Clinical SchoolUniversity of New South WalesSt George HospitalKogarahNSWAustralia; ^4^ Northern Sydney Local Health DistrictRoyal North Shore Hospital CampusSt LeonardsNSWAustralia; ^5^ Faculty of HealthUniversity of Technology SydneyUltimoNSWAustralia; ^6^ School of Nursing and MidwiferyMenzies Health Institute QueenslandGriffith UniversityNathanQldAustralia; ^7^ Department of Infection Control and Infectious DiseasesGold Coast University HospitalGold Coast Hospital and Health ServiceSouthportQldAustralia; ^8^ Centre for Quality and Patient Safety ResearchSchool of Nursing and MidwiferyDeakin UniversityBurwoodVicAustralia; ^9^ Midwifery Research CentreEastern HealthDeakin University NursingBox HillVicAustralia

**Keywords:** behaviour change, clinical practice, evidence based, evidence informed, implementation science, knowledge translation, nursing, research, trauma

## Abstract

**Aims and objectives:**

To describe the importance of, and methods for, successfully conducting and translating research into clinical practice.

**Background:**

There is universal acknowledgement that the clinical care provided to individuals should be informed on the best available evidence. Knowledge and evidence derived from robust scholarly methods should drive our clinical practice, decisions and change to improve the way we deliver care. Translating research evidence to clinical practice is essential to safe, transparent, effective and efficient healthcare provision and meeting the expectations of patients, families and society. Despite its importance, translating research into clinical practice is challenging. There are more nurses in the frontline of health care than any other healthcare profession. As such, nurse‐led research is increasingly recognised as a critical pathway to practical and effective ways of improving patient outcomes. However, there are well‐established barriers to the conduct and translation of research evidence into practice.

**Design:**

This clinical practice discussion paper interprets the knowledge translation literature for clinicians interested in translating research into practice.

**Methods:**

This paper is informed by the scientific literature around knowledge translation, implementation science and clinician behaviour change, and presented from the nurse clinician perspective. We provide practical, evidence‐informed suggestions to overcome the barriers and facilitate enablers of knowledge translation. Examples of nurse‐led research incorporating the principles of knowledge translation in their study design that have resulted in improvements in patient outcomes are presented in conjunction with supporting evidence.

**Conclusions:**

Translation should be considered in research design, including the end users and an evaluation of the research implementation. The success of research implementation in health care is dependent on clinician/consumer behaviour change and it is critical that implementation strategy includes this.

**Relevance to practice:**

Translating best research evidence can make for a more transparent and sustainable healthcare service, to which nurses are central.

**What does this paper contribute to the wider global clinical community?**

- Practical, evidence‐informed explanation and suggestion for knowledge dissemination and translation to clinical nursing practice.
- Methods to build knowledge translation into study design and conduct.
- Knowledge translation is not a linear procedure and involves many processes, systems and interactions of the researcher and knowledge users.
- Implementing evidence by translating knowledge needs planning and strategy that address the complexity of healthcare systems.

## Background and aim

The importance of robust scholarly research for quality, safe, effective and efficient care of patients and their families is well established (Australian Commission on Safety and Quality in Health Care 2009). Although research evidence is being produced at an increasing rate, change in clinical practice to reflect this evidence has lagged behind (Kitson 2008, Benner *et al*. 2010). For example, in Australia, clinician compliance with providing appropriate care for 22 conditions in large nationwide cross‐sectional study ranged from 32–86% (Runciman *et al*. 2012). In the United States, it is reported that <20% of what physicians do has solid research to support it (Kumar & Nash 2011). With more nurses in the frontline of health care than any other healthcare profession, nurse‐led research is increasingly recognised as a critical pathway to practical, effective and cost‐effective ways of reducing hospital errors, cutting down on unnecessary costs and improving patient outcomes (World Health Organization 2012). This practice paper aims to describe the importance of, and considerations for nurses to successfully disseminate and translate research into clinical practice.

## Design and method

This is a practice paper that interprets the knowledge translation literature for clinicians interested in conducting translational research and translating robust research evidence into clinical practice. The discussion is informed by the scientific literature around knowledge translation, implementation science and clinician behaviour change, and presented from the nurse clinician perspective. We discuss the importance of disseminating research and explain the definition and role of knowledge translation within the knowledge‐to‐action cycle. This is followed by practical, evidence‐informed suggestions to overcome the barriers and facilitate enablers of knowledge translation. Examples of nurse‐led research incorporating the principles of knowledge translation in their study design that have resulted in improvements in patient outcomes are provided. These examples are supported by a discussion of the supporting theories and evidence to maximise the opportunities and traction of the uptake of the evidence into practice.

## Discussion

### Disseminating research knowledge

Central to nurse‐led research and knowledge translation is dissemination. A research study is not complete until the study findings have been disseminated via presentations at professional forums and published in a peer‐reviewed journal and where appropriate recommendations regarding how the research findings could be translated into clinical practice are made. Research involves considerable intellectual, time and financial commitments by researchers, participants and funding organisations. It is often conducted using public funds under the guise of the common good. Consequently, researchers are obliged and required to share the findings of their project with others, regardless of the results. Moreover, the World Health Organization (WHO), in its position on Interventional Clinical Trial Results, states that it is unethical to conduct human research without publication and dissemination of the results of that research, as withholding results may subject future volunteers to unnecessary risk (Matosin *et al*. 2014). While there is a clear bias towards publication of positive response (Matosin *et al*. 2014), it is equally important to publish studies with negative or equivocal results for this reason. Furthermore, the WHO states that clinical trial results be submitted for publication in a peer‐reviewed journal within 12 months of study completion (Moorthy *et al*. 2015). Fortunately, there are many opportunities for sharing new knowledge, not only by writing for journals or books, but also by using social media, speaking at conferences and other events about the research outcomes. When planning research, it is critical to consider prospectively how findings will be disseminated and to be cognisant of this throughout the research process.

Successful dissemination and uptake of research evidence requires identifying the appropriate audience and tailoring messages via appropriate mediums. When analysing study data and interpreting the results, researchers must address the study aims and answer the research question(s) in view of the background research problem and its significance. The conduct of the research should also consider how the study findings should or could influence clinical practice, education, policy or future research. Such recommendations should inform dissemination activities. Targeted dissemination activities can include summaries for stakeholders, educational sessions with clinicians and/or policymakers, development and implementation of clinical guidelines and media engagement (Canadian Institutes of Health Research (CIHR) 2014; Table 1). At the heart of dissemination of research findings is knowledge translation.

**Table 1:** Choosing dissemination forums for research findings

*Study site*: Provide summary of results to key stakeholders at hospital level (such as nursing and midwifery executive, quality unit or nursing education), at unit level for distribution to clinical staff, present research findings at meetings or education sessions.
*Conference*: Choose best audience for the work, how to get funding to go to a conference (chose early bird rate, scholarships, industry sponsorship, special purpose funds, build into research grant).
*Journal*: Choose best audience for the work, review table of contents for best fit, seek advice, resources for writing for publication.
*Social media*: For example, Twitter^™^, LinkedIn^™^.
*Media*: Local newspaper, media release, hospital public relations, professional newsletters or magazines, research information dissemination organisations.
*Nursing organisations*: Specific to the type of research, for example the College of Emergency Nursing Australasia or the Society of Trauma Nurses.

### What is knowledge translation?

Knowledge translation is the process through which research knowledge is created, circulated and adopted into clinical practice. Synonymous terms are used by researchers around the world. A study involving 33 research funding agencies across nine different countries identified 29 different terms referring to knowledge translation (Graham *et al*. 2005). For example, similar processes are called *research utilisation* in the UK and Europe, *research dissemination, diffusion* or *knowledge uptake* in the USA, and *knowledge translation* and *knowledge‐to‐action* in Australia and Canada (Strauss & Corbin 1990, Graham *et al*. 2006). The Canadian Institute of Heath Research (CIHR) definition of knowledge translation is widely accepted and commonly cited in healthcare literature (Graham *et al*. 2006, Lang *et al*. 2007, Bjørk *et al*. 2013; Box 1). Knowledge translation is not simply a linear procedure but involves many processes, systems and interactions of the researcher and knowledge users. The level at which these interactions take place varies depending on the situation and application of knowledge.

Box 1 **Definition of Knowledge Translation**

*‘knowledge translation (KT) is defined as a dynamic and iterative process that includes synthesis, dissemination, exchange and ethically‐sound application of knowledge to improve the health of Canadians, provide more effective health services and products and strengthen the health care system*.

*This process takes place within a complex system of interactions between researchers and knowledge users which may vary in intensity, complexity and level of engagement depending on the nature of the research and the findings as well as the needs of the particular knowledge user’ (Canadian Institutes of Health Research (CIHR)* 2014 *)*.

### Can all research evidence be translated?

While all research should be disseminated, not all research is readily translatable. The design, applicability and strength of the research should be assessed, and the evidence for translation determined to be relevant and sound. This process is rarely simple, and the increasing volume of research evidence being produced, access to new evidence, the skills to appraise the quality of the evidence, time to locate and read evidence, and the capacity to apply evidence (Gravel *et al*. 2006) are some of the major barriers to dissemination and translation. Strategies to promote the use of research in practice by clinicians continue to be devised as the complexity of the application of evidence into practice has been recognised. One strategy now commonly used is *knowledge distillation*, that is the synthesis of findings from the most rigorous research available on a specific topic into systematic reviews and guidelines (Straus *et al*. 2009). The synthesis can then be presented to clinicians as practice guidelines or fact sheets (see, for instance, www.nhmrc.gov.au/guidelines/titles_guidelines.htm or www.clinicalguidelines.gov.au for two Australian organisations providing practice guidelines, https://www.nice.org.uk/guidance in the UK, http://www.ilcor.org/home/ for resuscitation, https://www.cma.ca/En/Pages/clinical-practice-guidelines.aspx in Canada). While sourcing sufficient evidence to base practice on is an ongoing challenge, so too is identifying established evidence and translating it into practice (Titler 2008). The lapse between the publication of evidence and its implementation into practice is referred to as an *evidence–practice gap* (National Institute of Clinical Studies 2003). Addressing this gap requires *knowledge translation*.

### Knowledge‐to‐action cycle

**Figure 1:**
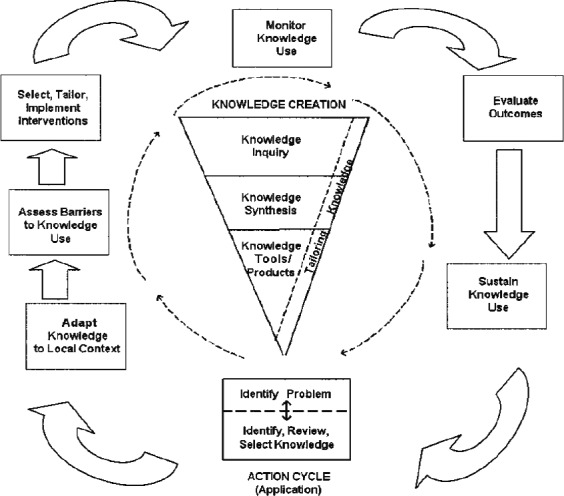
Knowledge‐to‐action cycle (Graham *et al*. 2006).

Knowledge translation forms part of the knowledge‐to‐action cycle (Fig. 1) (Graham *et al*. 2006). The knowledge‐to‐action cycle details the sequence and steps involved in achieving the transfer of research knowledge into clinical practice consisting of two phases. The initial creation phase consists of synthesising knowledge as part of producing new tools, such as clinical guidelines in response to an identified clinical problem. This step ensures knowledge is founded on the best available evidence prior to progressing to the action component, which is the process of implementing and evaluating new knowledge in clinical practice (Graham *et al*. 2006). The action cycle comprises seven phases: (1) identify problem and relevant research; (2) adapt research to local context; (3) assess barriers to using the knowledge; (4) select, tailor and implement interventions; (5) monitor knowledge use; (6) evaluate outcomes; and (7) sustain knowledge use.

### Getting traction in knowledge translation

Despite the importance of research knowledge translation, barriers to understanding, conducting, and evaluating evidence impede nurses’ uptake of research at an individual, unit and organisational level (Leasure *et al*. 2008). This was confirmed in a survey conducted by the Emergency Nurses Association (ENA) in the United States (Chan *et al*. 2011). Nine hundred and seventy‐eight ENA members completed a survey which assessed nurse's involvement and uptake in research and perceived barriers to research. At an individual level, it was found that nurses lacked knowledge about appraising research preventing them from implementing research knowledge in their clinical practice. At a unit level, barriers included lack of assistance from managers and colleagues in beginning a project or having the authority to implement change. Insufficient time provided by the organisation was also found to be an impeding factor failing to provide nurses with the support and time required to conduct research and change practice (Chan *et al*. 2011). Other studies have also identified that the attitudes and beliefs of nurses as obstacles to research being translated into nursing practice (MacDonald 2002, Brown & McCormack 2005, Davies *et al*. 2007, Newhouse 2007). The process through which individual attitudes and beliefs are formed, interest of administrators at a unit and organisational level must be addressed to promote research translation into clinical practice.

### Enablers to knowledge translation

There are many well‐documented enablers of successful knowledge translation. Translation of findings should be considered in initial study design and should be a major consideration when developing the study aim(s). When planning research, by prospectively thinking about how findings will be translated to clinical practice, key considerations may be what clinicians will need to change their behaviour and what resources the organisation will require to permanently maintain (sustain) the intervention. Further, it is essential to consider the ongoing monitoring and evaluation of the intervention to enable refinement and determination of impact (be that negative or positive). This process will ensure that data will be collected as part of the research to inform the implementation of the research findings if appropriate, for example times of day where problems occur or staff opinion about the proposed intervention. If applying for funding to conduct research, it is important to budget for implementation and evaluation of the proposed work.

The success of any implementing practice change is heavily reliant on senior clinician support (Bennetts *et al*. 2012), those that will be impacted by the intervention and those that will be required to act on the intervention (the end users). It is critical to involve end users throughout the research process. In our complex and multidisciplinary healthcare system, aside from our patients, who are at the centre of care, those impacted are likely to include multiple services and medical specialties from outside of the instigating department. This could include the hospital switch board, pathology, radiology, allied health, as well as hospital executive. Some suggestions for this multidisciplinary type implementation are establishment of a working party of key stakeholders to develop a consensus plan to streamline successful implementation. This process is likely to be facilitated if the instigator is an employee or has close links with staff at the target site. The key principles around incorporating translation into research design are summarised in Table 2 and an example in Table 3.

**Table 2:** Key principles to building knowledge translation into research design

1. Begin and plan with the end in mind
2. Produce evidence that is useful, not just interesting
3. Resource knowledge translation and exchange
4. Seek outcomes that will last
5. Involve end users throughout (Brand & Silburn 2014)

**Table 3:** Nurse‐led translational research example 1: Improving the emotional well‐being of major trauma patients (Wiseman *et al*. 2015, 2016)

*Aims*: To determine the incidence and predictors of depression, anxiety and stress (precursors to PTSD) in major trauma patients to inform an evidence‐informed programme for early intervention.
*Background*: Traumatic injury is a leading cause of psychological and physical disability across all age groups, responsible for 10·7% of the global burden of disease. Trauma patients report a substantial reduction in health‐related quality of life compared to other patients. Despite the known associations between injury, depression, anxiety, ASD and PTSD, prior to this project there was no known established routine screening tool for mental health outcomes in any Australian trauma centre. Further, there was no established referral process for those who do report symptoms of negative emotional responses after injury.
*Methods*: A 14‐month mixed methods study was conducted with 201 major trauma patients. Levels of depression, anxiety and/or stress symptoms were measured at baseline, three and six months using the DASS‐21. Patients reporting high levels were interviewed about their experiences and needs. Descriptive statistics and thematic analysis results were integrated. Then, in collaboration with multiple disciplines (allied health, mental health, trauma, GPs, nursing) and consumers, a sustainable, cost neutral screening, referral and improved discharge process for patients was implemented.
*Results*: In total, 54% of patients experienced high levels of depression, anxiety and/or stress symptoms in the six months following injury. Key qualitative findings were the extreme negative emotional responses experienced many months after the injury, reluctance to seek emotional support and a lack of emotional support provision by the health service. Since 2014, more than 2000 trauma patients have been telephoned within a week of their discharge from hospital by the hospital trauma nurses to enquire as to their physical and emotional well‐being and provide guidance. A total of 122 patients have been screened for symptoms of DAS when they return to the trauma clinic for review. Patients reporting symptoms are referred to their GP, clinical psychologists and if actively suicidal to the site acute mental health team. Guidance is provided to all patients on discharge on what to expect emotionally following injury and where they can seek help should they experience this. This process is being monitored.
*Conclusions*: Translation of findings resulted in implementation of an intervention that for the first time in Australia provides a clear process for the screening and referral of the injured patient in need of mental health support.

### Barriers to knowledge translation

There are also many well‐documented barriers to research translation. Multiple factors influence the uptake of research into practice. It is challenging to introduce and sustain evidence and evidence‐informed protocols in the context of competing priorities in health care. Despite high‐level recommendations to improve implementation of evidence‐based practice, implementation is variable. Numerous organisational and individual factors impact implementation and uptake, including clinician behaviour, lack of time, difficulties in developing evidence‐based or informed guidelines, a lack of continuing education and an unsupportive organisational culture (Haynes & Haines 1998, Wallis 2012), the availability and dissemination of evidence, individual motivation and the culture of specific healthcare practices (McKenna *et al*. 2004). Central to successful implementation of research evidence into clinical practice is changing human behaviour. Any attempt to improve the quality of care for patients by translating research must incorporate a clear understanding of the associated barriers to, and facilitators of, behaviour change. Understanding these is also fundamental to the development of a feasible, successful and sustainable implementation strategy.

### Theories to inform knowledge translation

A variety of models and theories have been developed in attempt to conceptualise the multifaceted process of knowledge translation. The transformation learning theory developed by Mezirow (Mezirow 1978, 2000, 2004) assists the process of knowledge translation through acknowledging the role and impact of attitudes and beliefs, which are constantly cited as barriers to research utilisation (MacDonald 2002, Brown & McCormack 2005, Davies *et al*. 2007, Newhouse 2007). To successfully engage clinicians and change their behaviours based on sound research knowledge, their attitudes and beliefs towards the proposed new knowledge must be learned, shaped and transformed (Matthew‐Maich *et al*. 2010). The clinicians existing thoughts and stances must be unlearned, and the new way of approach adopted. One such way to achieve this is to use tools to design implementation interventions using the theoretical domains framework (French *et al*. 2012) or the behaviour change wheel (Michie *et al*. 2011) discussed below. Further, knowledge translation requires design and implementation of interventions.

### Implementation science

Implementation is a science and can be encompassed within the normalisation process theory, which characterises implementation as a social process of collective action (May 2013). The intent of implementation science is to investigate and address major contextual factors (e.g. social, behavioural, economic, management) that hinder successful implementation, test new approaches and determine causal relationships (Fogarty International Center 2013). The Consolidated Framework for Implementation Research (CFIR) published in 2009 provides a pragmatic structure to promote verification about what works where and why across multiple contexts and includes five major domains: intervention characteristics, outer setting, inner setting, characteristics of the individuals involved and the process of implementation (Damschroder *et al*. 2009). This work provides a foundation for researchers implementing and evaluating knowledge translation to build the implementation knowledge base across multiple settings (Damschroder *et al*. 2009). As with all research, and to truly validate the CFIR and other research frameworks, descriptions must be precise enough to enable measurement and reproducibility (Proctor *et al*. 2013). When publishing research, researchers should clearly explain how they justified the selection of specific framework constructs, integrated the framework throughout the research process (in study design, data collection, and analysis) and link determinants of implementation to outcomes to contribute to this emerging field of research (Kirk *et al*. 2016).

### Planning for implementation

Implementing evidence by translating knowledge needs planning and strategy that address the complexity of healthcare systems, individual practitioners, managers (Titler 2008) and strong organisational support and patronage (Bate *et al*. 2008). There are multiple models available on which to develop and plan an implementation strategy (Schaffer *et al*. 2013). Perhaps the most well known in health is the Promoting Action on Research Implementation in Health Services Framework, or PARIHS Framework (Rycroft‐Malone 2004), which is a conceptual framework that suggests fundamental and interrelating elements that influence effective implementation of interventions. There is a need for this and other implementation models to undergo more robust evaluation of their effectiveness in use in implementation projects (Helfrich *et al*. 2010, Proctor *et al*. 2013).

Using a systematic four‐step approach as the principal framework to inform intervention development process is ideal. The four steps consist of questions to direct the choice of the most appropriate components of an implementation intervention (French *et al*. 2012) and can be iteratively adjusted and refined to suit other contexts. For example, the following steps recommended by French *et al*. (2012) in conjunction with the theoretical domains framework (Cane *et al*. 2012) have been evaluated as effective. It is also practical and pragmatic:

1. Who needs to do what, differently?
2. Using a theoretical framework, which barriers and enablers need to be addressed?
3. Which intervention components (behaviour change techniques and mode(s) of delivery) could overcome the modifiable barriers and enhance the enablers?
4. And how can behaviour change be measured and understood?

There are myriad templates available online to then guide the finer details of implementation plans, which all have common components. Many health services have a health redesign or implementation unit which may assist in the process, beginning by outlining the project purpose and justification (i.e. what will be used to introduce the plan to others). Many of these actions will have been made much smoother if the key stakeholders have been engaged in the process. It is also important to conduct a stakeholder needs analysis to identify the key stakeholders and their expectations and needs with respect to the project outcomes. The responsibilities for each person should then be established, alongside a communication strategy, and decisions on the interventions to be used to implement your evidence and timeline (Centre for Healthcare Redesign 2014).

### Implementation interventions to translate knowledge

There is a vast array of intervention techniques available to translate research‐based evidence into practice, for example visual cues (such as signs in the clinical area), audit, educational seminars, prompts, clinical guidelines, protocol and leadership involvement (Wuchner 2014). Interventions with the most likelihood of sustainable success are generally multimodal. To decide how to best to implement change depends on what you are trying to change. Changing behaviour is not simple, but is most effective if interventions are based on the principles of behaviour change, and knowing what it is exactly that you need to change. Three validated tools to use either in isolation or together are the theoretical domains framework (Cane *et al*. 2012), the behaviour change wheel (Michie *et al*. 2011) and the behaviour change technique taxonomy (BCTT; Michie *et al*. 2013), which are the specific behaviour change techniques to use in interventions focused on behaviour change.

Once it is determined who is going to need to change their behaviours, the theoretical domains framework (Cane *et al*. 2012) helps you to consider each of possible influences on behaviour in 14 domains including ‘knowledge’, ‘skills’, ‘beliefs about capabilities’, ‘optimism’, ‘beliefs about consequences’, ‘reinforcement’, ‘intentions’, attention and decision processes’, ‘environmental context and resources’, ‘social influences’ and ‘behavioural regulation’. For example, to determine what may need to be addressed to change clinician behaviour, a staff survey could be conducted with questions mapped to each of the domains. For example, ‘Do you think that the X protocol improves patient care?’ would be mapped to ‘beliefs about consequences’. If the majority of staff do not think that the protocol will deliver improved care, they may not make it a priority to change their behaviour, and you now know that this is an area that you need to address. But how to do it?

The behaviour change wheel and the BCTT are linked to the theoretical domains framework and will guide choice of interventions and techniques. For example, to address beliefs about consequence, the interventions known to do this are education, modelling and persuasion. There are multiple ways to educate, model and persuade. The BCTT provides a range to choose which would be suitable for the target site, staff and context. For example, a technique known to be effective in persuasion is having a senior, well‐respected clinician repeatedly model the behaviour you want the rest of the team to do. Using behaviour change techniques outlined in the BCTT also adds strength to your work because it means your work will be observable (people will know what you have done) and replicable.

### Implementation evaluation

Research utilisation implies not only the implementation of evidence into practice, but also the evaluation of consequent changes in practice (Jones 2000). It is no longer acceptable to implement a change in clinical care and not evaluate the impact of that change. That is, if the research evidence is applied in a given context, the resulting change should be evaluated in terms of the outcomes, considering patients, consumers, clinicians and the organisation. It is crucial to build implementation evaluation into study design by ensuring collection of data that will be able to be used to determine how well the intervention has been adopted, For example, Do all staff comply with the introduced protocol all the time? If they do (or do not), Why and what difference does this make? An example of this is demonstrated in Table 4. A summary of key knowledge translation terms is provided in Table 5.

**Table 4:** Nurse‐led translational research example 2 – Changing State‐wide Stroke Practice: The QASC Implementation Project (Middleton *et al*. 2011, 2015)

*Background*: The Quality in Acute Stroke Care (QASC) Trial (Middleton *et al*.) determined that a multidisciplinary supported, nurse‐initiated, evidence‐based intervention involving supported implementation of clinical protocols to manage fever, hyperglycaemia and swallowing (FeSS protocols) following stroke decreased death and dependency by 16% (*p* = 0·002); reduced temperatures (*p* = 0·001) and glucose levels (*p* = 0·02); and improved swallowing management (*p* = <0·001). Yet, upscale and spread of even proven interventions on a state‐wide level is challenging.
*Aim*: To implement the FeSS protocols from the QASC Trial in all 36 stroke services in NSW, Australia.
*Method*: The 14‐month translational project replicated the intervention from the original QASC Trial. The investigators conducted barrier and enabler assessments and an educational workshop, engaged local opinion leaders, used reminders and provided ongoing site champion support. Participating sites audited 40 pre‐ and 40 postimplementation medical records using the National Stroke Foundation clinical audit web‐based tool.
*Results*: All (*n* = 36, 100%) sites participated in the medical record audit (100% response rate) providing data for a total of 2144 patients (pre‐implementation: *n* = 1062; postimplementation: *n* = 1082). Significantly increased proportions of patients received care according to the fever (pre: 69%; post: 78%; *p* = 0·0031), hyperglycaemia (pre: 23%; post: 34%; *p* = 0·0085) and swallowing (pre: 42%; post: 51%; *p* = 0·0331) protocols postimplementation.
*Conclusion*: These results provide rare evidence of successful research translation of Class 1 Level B evidence across an entire state in a short time frame and in the real world of clinical practice.

**Table 5:** Key knowledge translation terms

*Evidence‐practice gap*: The lapse between the publication of evidence and its implementation into practice (National Institute of Clinical Studies 2003).
*Evidence informed*: The term ‘evidence informed’ versus the term evidence based is slightly different and oft a topic of debate. The difference between EB and EI is that EB is grounded in the demonstrated positive outcomes discovered through scientific research or rigorous evaluation. EI on the other hand, are guided by research, evaluation and clinical expertise (Sawatzky‐Dickson 2007, Canadian Nurses Association 2010). Evidence informed is the term used by WHO http://www.who.int/evidence/about/en/. Until the evaluation of guidelines implemented as a result of evidence occurs, evidence informed is the most appropriate term to use.
*Basic research*: ‘is performed without thought of practical ends. It results in general knowledge and an understanding of nature and its laws. This general knowledge provides the means of answering a large number of important practical problems, though it may not give a complete specific answer to any one of them. The function of applied research is to provide such complete answers’ (Bush 1945).
*Clinical research*: ‘Patient‐oriented research. Research conducted with human subjects (or on material of human origin such as tissues, specimens and cognitive phenomena) for which an investigator (or colleague) directly interacts with human subjects. Patient‐oriented research includes: (1) mechanisms of human disease, (2) therapeutic interventions, (3) clinical trials, or (4) development of new technologies. Epidemiologic and behavioral studies, outcomes and health services research’ (National Institutes of Health 2001)
*Translational research*: ‘Translational research fosters the multidirectional integration of basic research, patient‐oriented research, and population‐based research, with the long‐term aim of improving the health of the public. T1 research expedites the movement between basic research and patient‐oriented research that leads to new or improved scientific understanding or standards of care. T2 research facilitates the movement between patient‐oriented research and population‐based research that leads to better patient outcomes, the implementation of best practices, and improved health status in communities. T3 research promotes interaction between laboratory‐based research and population‐based research to stimulate a robust scientific understanding of human health and disease’ T4 Translation into the public sector (Rubio *et al*. 2010).
*Dissemination*: ‘A planned process that involves consideration of target audiences and the settings in which research findings are to be received and, where appropriate, communicating and interacting with wider policy and health service audiences in ways that will facilitate research uptake in decision‐making processes and practice’ (Wilson *et al*. 2010).
*Stakeholders*: ‘persons or groups that have a vested interest in a clinical decision and the evidence that supports that decision. Stakeholders may be patients, caregivers, clinicians, researchers, advocacy groups, professional societies, businesses, policymakers, or others. Each group has a unique and valuable perspective’ (Agency for Healthcare Research and Quality 2014).
*End users*: ‘The ultimate consumer of a product, especially the one for whom the product has been designed’ (The American Heritage Dictionary 2013).
*Implementation science*: ‘the scientific study of methods to promote the systematic uptake of research findings and other evidence‐based practices into routine practice, and, hence, to improve the quality and effectiveness of health services and care’ (Eccles & Mittman 2006).
*Behaviour Change interventions*: ‘coordinated sets of activities designed to change specified behaviour patterns. In general, these behaviour patterns are measured in terms of the prevalence or incidence of particular behaviours in specified populations (e.g., delivery of smoking cessation advice by general practitioners). Interventions are used to promote uptake and optimal use of effective clinical services, and to promote healthy lifestyles’ (Michie *et al*. 2011).

## Conclusion

Translating best research evidence can make for a more transparent and sustainable healthcare service, to which nurses are central. More importantly, the translation of evidence can bring about cultural, behavioural and practice change reducing the research–practice gap. Through the translation of evidence, patient safety and care responses can be recalibrated to optimise outcomes for patients and staff. Strong evidence must be translated into practice. Translation should be considered in research design, including the end users and an evaluation of the research implementation. The success of research implementation in health care is dependent on clinician or consumer behaviour change and it is critical that implementation strategy includes this.

## Contributions

KC led the development, writing and preparation of the manuscript. MF, RS and JC contributed, critically reviewed and prepared the manuscript for publication.

## Funding

Professor Curtis was supported by an NHMRC Translation of Research into Practice Fellowship (GNT 1067639).
